# Development and validation of a prediction tool to support engagement in HIV care among young people ages 10–24 years in Kenya

**DOI:** 10.1371/journal.pone.0286240

**Published:** 2023-06-30

**Authors:** Kate Wilson, Kawango Agot, Jessica Dyer, Jacinta Badia, James Kibugi, Risper Bosire, Jillian Neary, Irene Inwani, Kristin Beima-Sofie, Seema Shah, Nahida Chakhtoura, Grace John-Stewart, Pamela Kohler

**Affiliations:** 1 Department of Global Health, University of Washington, Seattle, WA, United States of America; 2 Impact Research and Development Organization, Kisumu, Kenya; 3 Department of Epidemiology, University of Washington, Seattle, WA, United States of America; 4 University of Nairobi/Kenyatta National Hospital, Nairobi, Kenya; 5 Northwestern University Medical School/Bioethics Program at Lurie Children’s Hospital, Chicago, IL, United States of America; 6 Eunice Kennedy Shriver National Institute of Child Health and Human Development, National Institutes of Health, Washington, DC, United States of America; 7 Department of Medicine, University of Washington, Seattle, WA, United States of America; 8 Department of Pediatrics, University of Washington, Seattle, WA, United States of America; 9 Department of Child, Family, Population Health Nursing, University of Washington, Seattle, WA, United States of America; Emory University School of Medicine, UNITED STATES

## Abstract

**Introduction:**

Loss to follow-up (LTFU) among adolescents and young adults living with HIV (AYALWH) is a barrier to optimal health and HIV services. We developed and validated a clinical prediction tool to identify AYALWH at risk of LTFU.

**Methods:**

We used electronic medical records (EMR) of AYALWH ages 10 to 24 in HIV care at 6 facilities in Kenya and surveys from a subset of participants. Early LTFU was defined as >30 days late for a scheduled visit in the last 6 months, which accounts for clients with multi-month refills. We developed a tool combining surveys with EMR (‘survey-plus-EMR tool’), and an ‘EMR-alone’ tool to predict high, medium, and low risk of LTFU. The survey-plus-EMR tool included candidate sociodemographics, partnership status, mental health, peer support, any unmet clinic needs, WHO stage, and time in care variables for tool development, while the EMR-alone included clinical and time in care variables only. Tools were developed in a 50% random sample of the data and internally validated using 10-fold cross-validation of the full sample. Tool performance was evaluated using Hazard Ratios (HR), 95% Confidence Intervals (CI), and area under the curve (AUC) ≥ 0.7 for good performance and ≥0.60 for modest performance.

**Results:**

Data from 865 AYALWH were included in the survey-plus-EMR tool and early LTFU was (19.2%, 166/865). The survey-plus-EMR tool ranged from 0 to 4, including PHQ-9 ≥5, lack of peer support group attendance, and any unmet clinical need. High (3 or 4) and medium (2) prediction scores were associated with greater risk of LTFU (high, 29.0%, HR 2.16, 95%CI: 1.25–3.73; medium, 21.4%, HR 1.52, 95%CI: 0.93–2.49, global p-value = 0.02) in the validation dataset. The 10-fold cross validation AUC was 0.66 (95%CI: 0.63–0.72). Data from 2,696 AYALWH were included in the EMR-alone tool and early LTFU was 28.6% (770/2,696). In the validation dataset, high (score = 2, LTFU = 38.5%, HR 2.40, 95%CI: 1.17–4.96) and medium scores (1, 29.6%, HR 1.65, 95%CI: 1.00–2.72) predicted significantly higher LTFU than low-risk scores (0, 22.0%, global p-value = 0.03). Ten-fold cross-validation AUC was 0.61 (95%CI: 0.59–0.64).

**Conclusions:**

Clinical prediction of LTFU was modest using the surveys-plus-EMR tool and the EMR-alone tool, suggesting limited use in routine care. However, findings may inform future prediction tools and intervention targets to reduce LTFU among AYALWH.

## Introduction

Adolescents and young adults living with HIV (AYALWH) in sub-Saharan Africa continue to experience lower retention in care and poorer outcomes than adults, despite the availability of antiretroviral therapy (ART) to improve individual health and reduce transmission risk [1]. Once treatment has been initiated, loss-to-follow-up (LTFU) among AYALWH ages 10 to 24 ranges from 20–30% depending on the population and outcome definition [2–4]. In Kenya, a country with a high burden of HIV, adolescents and young adults comprise nearly 50% of new HIV infections [5] and LTFU has been reported between 15–50% [6, 7]. LTFU can increase the risk of viral non-suppression through interruption of ART [8, 9]. An analysis of the population-based Kenya AIDS Indicator Survey data (2012) of factors associated with community viral load prevalence in Kenya found that younger age (15–29 years versus 30–64) and being out of HIV care (LTFU or never enrolled) were associated with detectable viral load (≥500 copies/ml) [10]. Recently, UNAIDS announced a renewed commitment to improve retention in care among AYALWH as a key strategy to end AIDS by 2030 [11].

Several factors can increase risk of LTFU from care among AYALWH [12]. These include HIV-related stigma [13], lack of ‘youth friendly’ providers or spaces [14, 15], lack of support transitioning to adult care [15], and depression [16]. Potential interventions to reduce risk of LTFU among AYALWH [17, 18] include family-based economic support [19–21], ‘youth-friendly’ services [14] and peer-support groups [22]. However, these interventions require significant resource investments for already strained health systems, and there is a need to develop approaches to prioritize which interventions to provide AYALWH when resources are scarce [23].

Many countries in sub-Saharan Africa have adopted differentiated service delivery models, a form of client-centered care intended to improve system efficiencies, quality, and outcomes [23]. Clients identified as clinically stable can shift to less frequent visits and multi-month ART refills, while unstable clients continue with standard care. In Kenya, AYALWH ages 20 and older are eligible for differentiated care, including multi-month ART refills and longer time between visits [24]. The emphasis on differentiated care prioritizes individuals doing well in care, however, there remains a need for guidelines to systematically improve care for those who are at risk for LTFU or who are unstable in care.

Clinical prediction tools are an effective, data-driven strategy to improve care and treatment decisions for a range of health conditions [25–31] including HIV [28, 31–33]. They are developed using data from similar client populations and adapted to work in a routine care setting [29, 34]. With expanded use of digital systems, there are new opportunities to use prediction tools to support HIV services using data from electronic health records systems [35, 36]. Clinical prediction tools have been developed to identify women who may be eligible for Pre-Exposure Prophylaxis (PrEP) services [27, 37, 38], adults in need of HIV testing [33], and adults at risk of viral failure who would benefit from adherence support [31, 39]. To date, there are no prediction tools designed to identify AYALWH at risk of LTFU.

A clinical prediction tool to identify AYALWH at risk of LTFU could support clinicians to better allocate intensified care to at-risk AYALWH before they are lost and to identify stable AYALWH for differentiated services. To address this gap, we developed and validated a clinical prediction tool to identify AYALWH at risk of LTFU using surveys and routine data.

## Materials and methods

### Setting and population

We conducted a prospective cohort study among AYALWH enrolled in care at six facilities in Kisumu and Homa Bay counties in Kenya. Selection criteria for the facilities were having an active electronic medical records (EMR) system, at least 100 AYALWH enrolled in care, and permission from facility managers. Data sources for this clinical prediction tool included AYALWH EMR and surveys. EMR data included all AYALWH ages 10 to 24 years enrolled in HIV care from October 1, 2018, until administrative censoring on February 29, 2020, due to COVID-19. Starting in April 2019, all eligible AYALWH ages 10 to 24 enrolled in care at the time were invited to participate in a cohort study that included behavioral surveys at enrollment and every 6 months. Study staff obtained written informed consent from AYALWH ages 18 to 24 or caregiver consent and adolescent assent for adolescents ages 10 to 17 years. Study staff administered face-to-face surveys during routine clinic visits in the participants’ preferred language (Kiswahili, Dholuo, or English).

### Outcomes and predictors

The primary outcome of the prediction tool was loss to follow-up, defined as >30 days late for a scheduled visit in the EMR during a 6-month period. We chose this definition to measure early risk of LTFU among AYALWH when interventions to support clinic attendance may be most effective [39]. Counting from last scheduled visit rather than last actual visit accounted for AYALWH who were on multi-month dispensing (MMD) schedules. For example, a client on a 3-month refill regimen would be LTFU if they were more than 30 days late a quarterly visit (4 months out of care). AYALWH were excluded if their first visit in the EMR occurred within 30 days of February 29, 2020, or if they had an enrollment survey that could not be linked to their EMR.

**Fig 1:**
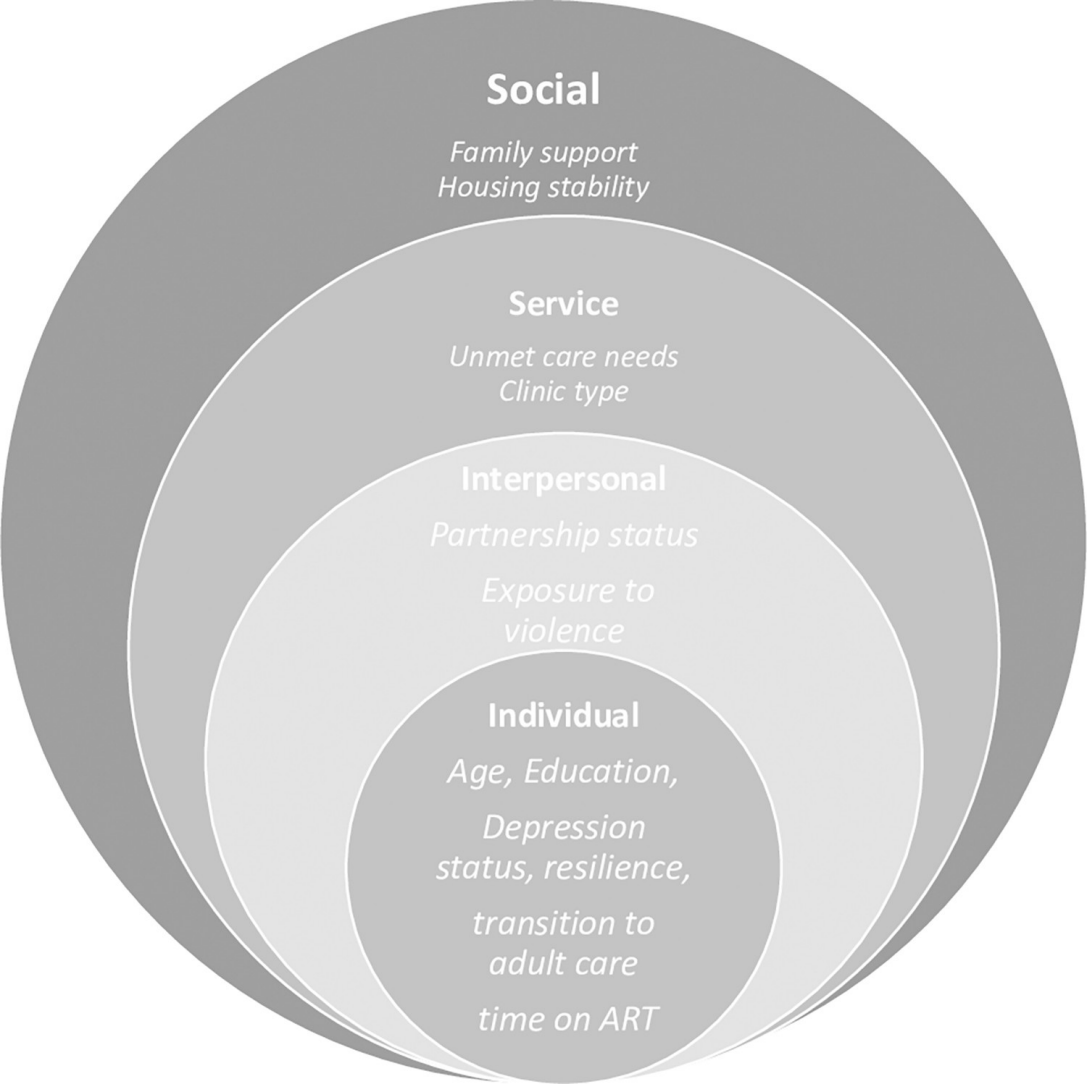
Adapted socio-ecological framework of potential domains and example predictors of loss to follow-up among adolescents and young adults with HIV.

Candidate predictors of LTFU were selected from AYALWH surveys and EMR data based on plausibly and prior studies among AYALWH [6, 13, 14]. We used an adapted ecological framework [40] to guide our thinking, and present example predictors in Fig 1.

Variables from surveys included social (pays own expenses), service (any caregiver accompaniment, any unmet clinical needs, peer support group attendance, use of any differentiated ART refill models, seen at youth-friendly vs. adult clinic), interpersonal (exposure to any physical, sexual, or emotional violence), and individual characteristics not captured in EMR (e.g. alcohol use, other drug use, did not complete secondary school). The variable *unmet clinical needs* was computed as indicating at least one need was not met after reading aloud a list of standard services that should be offered at the clinic visit, including contraception and pregnancy information, screening for STIs, depression, gender-based violence (GBV), and substance use, nutrition assessment, and referral to peer support groups. Asking clients whether their clinical needs were met at the visit is often used to evaluate quality of care [41]. Resilience was defined using a 2-item Connor-Davidson Resilience Scale [42]. Depressive symptoms were measured using Patient Health Questionnaire-9 (PHQ-9), social support by the Multidimensional Scale of Perceived Social Support (MSPSS) [43], alcohol use by the Alcohol Use Disorders Identification Test-Consumption (AUDIT-C) [44], and exposure to any physical, sexual, or emotional violence in the last six months using an adapted version of the World Health Organization (WHO) Violence Against Women survey [45]. Assessment of depressive symptoms using PHQ-9 score, substance use, exposure to recent violence, and participation in peer support group attendance are recommended for AYALWH in Kenya, although these data are not yet routinely incorporated in EMR systems [33, 36]. Candidate predictors available in EMR were age at first visit since October 1, 2018, marital status (single, married, divorced/widowed), WHO stage (recoded as a binary variable: 3 or 4, ‘advanced disease’ versus 1 or 2, ‘asymptomatic/early stage infection’) [46], transfer status (transferred in vs. never transferred), and time on ART. We created variables for newly enrolled in care (less than 6 months versus 6 months or more) and time on ART (less than 3 years versus 3 or more) from existing data. We used enrollment values of all predictors except for time on ART and WHO status, which were time-varying in the EMR. We computed time on ART as the total days between date of ART initiation until the outcome occurred or administrative censoring on February 29, 2020, converted to years. We used the first non-missing value for WHO stage after entry into the analysis cohort from each client record.

We developed two versions of the tool to predict high, medium, and low risk of LTFU. The ‘survey-plus-EMR’ tool included data among the subset of AYALWH who were enrolled in the cohort study and had surveys linked to EMR data (April 2019 to February 2020). We also developed an EMR-alone tool that included a larger sample of AYALWH in care (October 2018 to February 2020), some of whom were not enrolled in the cohort study therefore lacked survey data. We used methods for prediction rules [27, 28] and from risk scores used to predict HIV infection among women in Kenya [28] and South Africa [37] and virologic failure among adults living with HIV in Haiti [31]. Continuous variables and categorical variables with three or more levels were transformed into binary values. These included age group (adolescents as 10–19 and young adults as 20–24 years), marital status (single vs. ever married), PHQ-9 (0–4 vs 5 or higher), and AUDIT-C (0–3 vs 4 or higher).

Univariable Cox regression models were developed to estimate hazard ratios (HR) and 95% Confidence Intervals (95% CI) and two-sided α = 0.05. All regression models accounted for variability between facilities (e.g. size, volume, location) by clustering on facility. To evaluate potential effect modification by age group, all models were re-run with an interaction term between the predictor and binary age. We proposed developing age-group stratified prediction tools if the p-values of most interaction terms were <0.1. Since this was not the case, age group was included as a pre-specified predictor in the model. All predictors with significance level of 0.1 or less were included in multivariable regression models. We did not adjust for multiple comparisons to minimize loss of power. In this complete-case analysis, only AYALWH with non-missing data for all predictors were included. Overall, 70 individuals were dropped from the multivariable analyses for the survey-plus-EMR tool due to missing one or more responses, 41 from the training dataset and 29 from the validation dataset. We used Akaike Information Criteria (AIC) backwards elimination to determine the most predictive variables to include in the final model. If key variables were necessary to make the predictive model robust, these were retained in the model. Because WHO stage had >10% missing data, that variable was excluded from multivariable analysis, and all cases with missing WHO would have been dropped. We decided not to perform multiple imputation for variables with missing data to preserve the dataset as it would be used in routine care, where imputation is not conducted. Age at ART initiation was excluded due to collinearity with age group. A score was assigned to each predictor from the final stepwise regression model by dividing the coefficient for each predictor by the smallest coefficient among all predictors in the model and rounding to the nearest integer [46].

Tool performance was assessed through model testing according to standard steps for prediction tool development [46, 47]. Specifically, we calculated area under the receiver operating curve (AUC, and range 0–1.0) which is identical to the c-statistic for continuous outcomes [29] using optimal cut points of a binary version of the score to assess ability to differentiate between individuals at high and low risk of LTFU. We used the standard cut off AUC ≥0.7 for ‘good’ performance, and AUC of 0.60–0.69 for modest performance [29, 48]. We used a Brier score (range 0–1.0) to estimate the accuracy to predict LTFU. We then created a three-level score and assigned numeric values to the low, medium, and high-risk categories based on the score distribution in tertials. Each tool was developed in a random sample of 50% of the data (‘training dataset’) and externally validated in the remaining 50% of the data (‘validation dataset’) that was not used to generate the initial score. We validated the score using unadjusted Cox regression models in the validation cohort, accounting for clustering by facility. We used unadjusted analyses to develop a tool that providers could understand and explain to clients. We used the Global Wald test of significance for the risk score, against the null hypothesis that none of the risk levels in the score were different than zero. We also conducted 10-fold cross-validation procedures in the full dataset to evaluate the generalizability of each tool, estimated by AUC. We compared the ‘survey-plus-EMR’ tool with the EMR-alone tool descriptively to determine whether the EMR tool was able to provide comparable clinical prediction. All analyses were conducted in Stata 16.0 (College Station, Texas).

### Sample size and power

We estimated the minimal detectable hazard ratios of LTFU assuming sample size of 1,350 AYALWH enrolled on the cohort with EMR data and a LTFU proportion of 25% for an effective analytic sample of ~1,000. We estimated the minimum detectable difference in LTFU proportion at 80% power for a range of predictor levels (10%-50%), at α = 0.05, for the training and validation cohorts (n = 500 each). Under these assumptions, we estimated 80% power to detect RR ≥1.66 for common exposures and ≥2.12 for rare exposures in the training or validation samples, respectively.

This study was approved by the University of Washington Institutional Review Board and Maseno University Ethics Review Committee. We received permissions from County and health facility leadership prior to accessing EMR and viral load data.

## Results

### Survey-plus-EMR prediction tool

In the six facilities, 973 AYALWH had enrolled in the cohort study at the time of the analysis, and 108 did not have a linked EMR, resulting in 865/973 (88.9%) eligible individuals. Overall, 6-month LTFU was 19.2% and similar between the training and validation cohorts (88/433, 20.3% vs. 78/432, 18.1%, p = 0.40) (Table 1).

**Table 1:** Characteristics of AYALWH^a^ in the training and validation cohorts for the survey-EMR^b^ and EMR^b^-alone tools.

Survey plus EMR^b^ dataset (N = 795)	Training	Validation	p-value*
**Characteristic**	(n = 432)	(n = 433)	
Age			
10–19	324 (75.0)	331 (76.4)	- -
20–24	108 (25.0)	102 (23.6)	0.62
Female	274 (63.4)	284 (65.6)	0.51
Pays most of own expenses (n = 860)	25 (5.8)	23 (5.4)	0.77
Left school before age 18 (n = 858)	108 (25.2)	95 (22.1)	0.30
Mean social support score (n = 808)	3.5 (3.1, 4.0)	3.4 (3.0, 3.8)	0.17
Mean resilience score (n = 840)	3 (2, 3.5)	3 (2, 3.5)	0.58
Mild/moderate depressive symptoms^c^ (n = 802)	74 (18.7)	77 (18.9)	0.95
Harmful alcohol use^d^	8 (1.9)	1 (0.2)	0.02
Any drug use besides alcohol (vs. never) (n = 853)	4 (0.94)	10 (2.3)	0.11
Exposure to any violence in last 6 months	84 (19.4)	61 (14.1)	0.04
Self-reported in high-risk population	62 (14.4)	55 (12.7)	0.48
Knows their own HIV status	394 (91.2)	389 (89.8)	0.40
Would be ashamed if a family member had HIV (agree) (n = 851)	76 (17.8)	64 (15.1)	0.52
Would be ashamed if I had HIV (agree) (n = 852)	76 (17.9)	88 (20.6)	0.33
People should be ashamed for having HIV (n = 855)	35 (8.2)	42 (9.8)	0.15
Caregiver accompanied to visit	101 (23.5)	116 (26.9)	0.26
Not enrolled in a peer support group (n = 857)	230 (53.7)	219 (51.1)	0.43
At least 1 unmet clinical need	208 (48.2)	216 (49.9)	0.61
Seen at youth or pediatric clinic (vs adult) (n = 859) LTFU^e^	147 (34.4) 78 (18.1)	145 (33.6) 88 (20.3)	0.83 0.40
**EMR**^**b**^ **dataset (N = 2,696)**	* *	* *	* *
Age group (years)			
10–19	732 (54.3)	709 (52.6)	- -
20–24	617 (45.7)	638 (47.4)	0.40
Female	956 (70.9)	952 (70.7)	0.91
Ever married or partnered	456 (41.8)	507 (45.6)	0.08
In care ≥6 months	1,044 (77.4)	1,084 (80.5)	0.05
Transferred in	203 (15.1)	211 (15.7)	0.66
On ART^f^ < 3 years (n = 2,669)	637 (47.8)	626 (46.9)	0.66
WHO Stage 1 or 2 (n = 2,188)	918 (85.9)	943 (84.3)	0.30
Age at ART^f^ initiation (years) LTFU^e^	15.6 (8.0, 20.7) 370 (27.4)	15.8 (8.0, 20.5) 400 (29.7)	0.90 0.20

^a^ AYALWH: adolescents and young adults living with HIV

^**b**^ EMR: electronic medical records

^c^ measured using Patient Health Questionnaire-9 score ≥5

^d^ evaluated by the Alcohol Use Disorders Identification Test-Consumption >3

^e^ LTFU: lost to follow-up

^f^ ART: antiretroviral treatment

*P-values from Chi-square tests of proportions or t-tests of means.

In the training dataset of 432 AYALWH, we evaluated social, service, interpersonal (partner violence), psychosocial, and individual-level variables from surveys as candidate predictors of LTFU in univariable analysis (Table 2). Variables from EMR were age group, sex, marital status, newly enrolled, transfer status, time on ART, and WHO stage. Of the variables tested, PHQ-9 score, any unmet service need, no participation in a peer support group, enrollment in adult care, and newly enrolled were independently significantly associated with LTFU and evaluated in multivariable analyses (N = 795).

**Table 2:** Univariable and multivariable results of prediction modeling in the survey plus EMR^a^ and EMR^a^-alone tools.

	Univariable model		Full multivariable model			Stepwise multivariable analysis (AIC)^f^			Score
Characteristic	Univariable β (95%CI)	p-value	Adjusted β (95%CI)	Adjusted HR^g^ (95%CI)	p-value	Adjusted β (95%CI)	Adjusted HR^g^ (95%CI)	p-value	
**Survey plus EMR**^**a**^ **(N = 391)**									
Age group 20–24 (vs 10–19)	0.11 (-0.45–0.67)	0.70	-0.26 (-0.70–0.17)	0.77 (0.50–1.18)	0.23				
Female	0.06 (-0.40–0.53)	0.78	--	--					
Pays most of own expenses (n = 860)	0.69 (-0.15–1.54)	0.11	--	--					
Left school before age 18	-0.27, -0.89–00.35	0.39	--	--					
Mean social support score (n = 401)	-0.21, (-0.42–0.00)	0.06	--	--					
Mean resilience score (n = 419)	0.09 (-0.15–0.33)	0.45	--	--					
Mild/moderate depressive symptoms^b^	0.55 (0.10–1.00)	0.02	0.50 (0.06–0.94)	1.66 (1.07–2.57)	0.03	0.49 (0.05–0.92)	1.63 (1.05–2.51)	0.03	2
Harmful alcohol use^c^	-0.36 (-1.38–0.65)	0.48	--	--					
Any drug use besides alcohol	-0.63 (-2.51–1.26)	0.52	--	--					
Exposure to any violence in last 6 months (n = 432)	-0.40 (-1.13–0.35)	0.30	--	--					
Self-reported in high-risk population	-0.09 (-0.82–0.64)	0.81	--	--					
Knows their own HIV status (n = 431)	0.38 (-0.77–1.53)	0.52	--	--					
Would be ashamed if a family member had HIV (agree) (n = 426)	-0.21 (0.55–0.13)	0.24	--	--					
Would be ashamed if I had HIV (agree) (n = 424)	0.09 (-0.30–0.47)	0.66	--	--					
People should be ashamed for having HIV (n = 427)	-0.01 (-0.49–0.47)	0.97	--	-					
Caregiver accompanied to visit	-0.63, (-1.63–0.37)	0.22	`--	`--					
Not enrolled in a peer support group (n = 428)	0.52 (-0.07–1.11)	0.09	0.37 (-0.29–1.04)	1.45 (0.75–2.82)	0.27	0.37 (-0.26–0.99)	1.44 (0.77–2.68)	0.25	1
At least 1 unmet clinical need	0.33 (0.00–0.66)	0.05	0.39 (0.09–0.69)	1.47 (1.09–1.99)	0.01	0.32 (0.02–0.63)	1.39 (1.02–1.88)	0.04	1
In care ≥6 months	0.36 (-0.21–0.93)	0.22	--	--					
Transfer in (yes)	0.23 (-0.76–1.22)	0.64	--	--					
On ART^d^ < 3 years (n = 430)	0.08 (-0.61–0.76)	0.83							
Ever partnered (n = 342)^e^	0.31 (-0.01–0.63)	0.05	--	--					
WHO Stage 1 or 2 (n = 283)	0.001 (-0.26–0.26)	0.99	--	--					
Age at ART^d^ initiation	0.02 (-0.03–0.08)	0.40	--	--					
**EMR**^**a**^ **only tool (N = 1,349)**									
Age group 20–24 (vs 10–19)	0.36 (-0.17–0.90)	0.18	0.29 (-0.19–0.77)	1.34 (0.83–2.17)	0.24	0.29 (-0.19–0.77)	1.34 (0.83–2.17)	0.24	1
Female	0.08 (-0.26–0.41)	0.66	`--	`--		`--	`--		
In care ≥6 months	0.40 (-0.02–0.81)	0.06	0.30 (-0.04–0.65)	1.35 (0.96–1.91)	0.08	0.30 (-0.04–0.65)	1.35 (0.96–1.91)	0.08	1
Transfer in (yes)	0.11 (-0.20–0.42)	0.50	`--	`--					
On ART^d^ <3 years (n = 1,334)	0.25 (-0.21–0.71)	0.28	`--	`--					
Ever married or partnered (n = 1,090)	0.14 (-0.05–0.34)	0.14	`--	`--					
WHO Stage 1 or 2 (n = 1,069)	`-0.36 (-0.67- -0.04)	0.03	`--	`--					
Age at ART^d^ initiation (years) (n = 1,347)	0.04 (-0.01–0.09)	0.14	`--	`--					

^a^ EMR: electronic medical records

^b^ measured using Patient Health Questionnaire-9 score ≥5

^c^ evaluated by the Alcohol Use Disorders Identification Test-Consumption >3

^d^ ART: antiretroviral treatment

^e^ marital status dropped from the model because of >10% missing

^f^ Stepwise models included variables with lowest Akaike Information Criteria (AIC)

^g^ HR: Hazard Ratios from Cox regression modeling.

**Fig 2:**
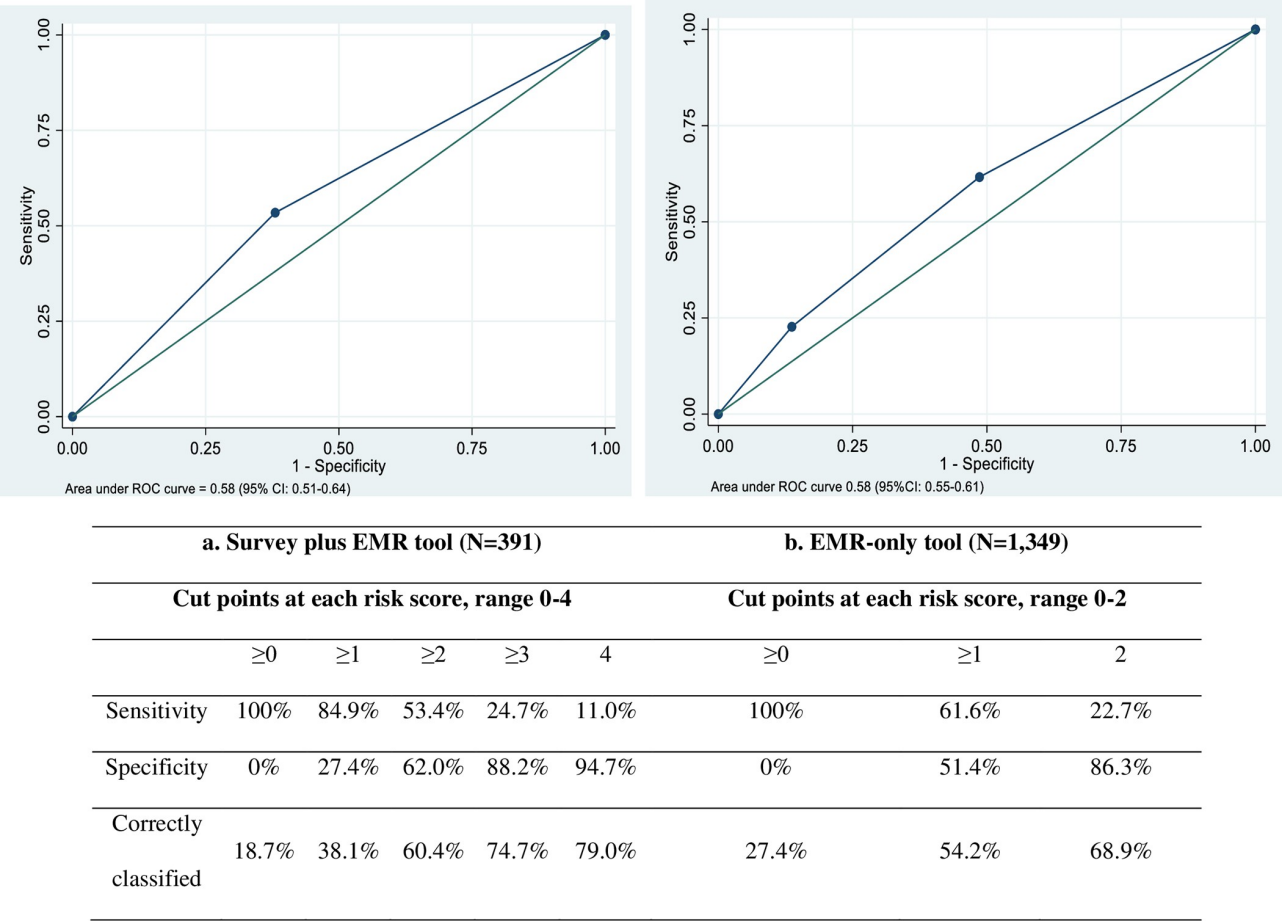
Area under the curve. a. Survey plus EMR (N = 391); b. EMR alone (N = 1,349).

Variables most predictive of LTFU in the final model using AIC selection were PHQ-9 score, no participation in a peer support group, and at least one unmet service need (lowest AIC = 837.29, partial log likelihood = —415.61, k = 3 parameters) (Table 3). Including age group in the model did not improve model performance. Total prediction scores ranged from 0 to 4. Using a 0.5 cut point for the binary form of the score, the AUC was 0.58 (95% CI: 0.51–0.64, standard error = 0.04), with sensitivity of 0.53 and specificity of 0.62, and Brier score of 0.40 (Table 3 and Fig 2A). The three-level prediction tool classified 15.9% of AYALWH as high risk, 25.6% medium risk, and 59.1% as low risk of LTFU, with medium- and high-risk scores associated with significantly greater risk of actual LTFU (medium risk, HR 1.52, 95%CI: 0.93–2.49, high risk, HR 2.16, 95%CI: 1.25–3.73; Global Wald Chi-square = 7.72 and p-value = 0.02 comparing medium and high to low-risk scores, degrees of freedom = 2).

**Table 3:** Survey plus EMR^a^ and EMR^a^-only tool predictor, score and performance.

Predictor			% LTFU^d^ (n = 73)	Score per predictor	Risk level (total score)	n (%) in each level			n (%) LTFU^d^ by risk level	
Survey plus EMR^a^ (n = 391)^b^										
Support group			27 (15.0)	0	High (3 or 4)	62 (15.9)			18 (29.0)	
No support group			46 (21.8)	1						
PHQ-9^c^ score 0–4			53 (16.7)	0	Medium (2)	98 (25.1)			21 (21.4)	
PHQ-9^c^ score 5+			20 (27.4)	2	Low (0 or 1)	231 (59.1)			34 (14.7)	
No unmet clinical needs ≥1 unmet clinical need			32 (15.4)	0						
			43 (20.7)	1						
Performance of the binary form of score^f^										
Brier score	0.40			range 0–1	Sensitivity	0.53	Cut point ≥1.5*			24.4 (39/160)
AUC^e^, 95% CI	0.58 (0.51–0.64)			range 0–1	Specificity	0.62	Cut point <1.5			14.7 (34/231)
**EMR** ^**a**^ **-only tool (N = 1,349)**										
*Age in years* 10–19 20–24		168 (23.0) 202 (32.7)		0	High (2)	218 (16.2)			84 (38.5)	
				1	Medium (1)	486 (36.0)			144 (29.6)	
Not new		260 (24.9) 110 (36.1)		0	Low (0)	645 (47.8)			142 (22.0)	
Newly enrolled				1						
Performance of the binary form of score										
Brier score		0.46		range 0–1	Sensitivity	0.62		Cut point ≥0.5		32.4 (228/704)
AUC^e^, 95% CI		0.58 (0.55–0.61)		range 0–1	Specificity	0.51		Cut point <0.5		22.0 (142/645)

^a^ EMR: electronic medical records

^b^ 38 of 433 AYALWH had missing PHQ-9 scores and 4 had missing responses to peer group enrollment and were excluded from the analysis

^c^ PHQ-9: Patient Health Questionnaire-9

^d^ LTFU: lost to follow-up

^e^ AUC: area under the curve

^f^AUC and Brier scores assessed using the prediction tool with a binary score of <1.5 and 1.5–4.0.

In the validation cohort (n = 404), medium (HR 1.58, 95%CI: 1.07–2.33) and high-risk scores (HR 1.46, 95%CI: 0.76–2.81) predicted greater risk of LTFU compared to low-risk scores and had a trend for an association (Global Wald Chi-square = 5.29 and p-value = 0.07, degrees of freedom = 1). There was limited generalizability using 10-fold cross validation in the full sample (AUC = 0.66, 95%CI: 0.63–0.72, standard error = 0.025, Chi-square = 11.72, p-value<0.001).

### EMR-alone prediction tool

A total of 2,696 records from six facilities were included in this analysis. Most AYALWH were ages 10–14 (28.2%) or 20–24 years (46.6%), female (70.8%), had been in HIV care at least 6 months (78.9%), and WHO stage 1 or 2 (85.1%). The median time on ART was 3.1 years (interquartile range [IQR] 0.1–7.6). Overall, 6-month LTFU in the EMR cohort was 28.6% (n = 770), with no significant difference between training and validation cohorts (27.4% vs. 29.7%, p = 0.20) (Table 1). The training dataset included 1,349 records. In the univariable analyses, we evaluated age group, marital status, sex, age at ART initiation (continuous), newly enrolled, transfer status, time on ART, and WHO stage as predictors of LTFU (Table 2). WHO status and newly enrolled were associated with significantly greater likelihood of LTFU in univariable analyses. Age group was retained because it improved model performance.

The final predictive model using AIC included age group and newly enrolled status (lowest AIC = 5518.17, partial log likelihood = -2592.085, k = 2 parameters). Individual scores ranged from 0 (low risk) to 2 (high risk) (Table 2). The prediction tool performance using an optimal cut point of 0.5 was poor and similar to the survey-plus-EMR tool. The AUC was 0.58 (95%CI: 0.55–0.61, standard error = 0.16), and sensitivity and specificity were 0.62 and 0.51, respectively (Table 3 and Fig 2B). As a three-level score, there was a stepwise trend of higher risk of LTFU with higher scores. When the prediction tool was evaluated in the validation cohort (n = 1,347), medium (HR 1.65, 95%CI: 1.00–2.72) and high-risk scores (HR 2.40, 95%CI: 1.17–4.96) predicted significantly higher LTFU compared to low-risk scores (Global Wald Chi-square = 6.84 and p-value = 0.03). In 10-fold cross validation, the prediction tool had an AUC of 0.61 (95% CI:0.59–0.63, standard error = 0.0120, Chi-square = 14.04, p-value<0.001).

## Discussion

We developed two clinical prediction tools, one using EMR data combined with survey data and one using only EMR data, to predict LTFU among AYALWH and found similarly poor predictive performance. Overall, both tools showed poor performance by standard measures. The survey-plus-EMR tool, which included PHQ-9 score, peer support group enrollment, and any unmet care needs had slightly better performance (AUC) than the EMR-alone tool in the training dataset. The EMR-alone tool, which only included age and time in care, predicted LTFU in the validation cohort. The tools had similar generalizability when cross-validated in the full samples. While both tools demonstrated moderate predictive ability, results may guide future clinical prediction tools and decision-support interventions with AYALWH.

We chose the outcome of early LTFU to support early intervention before AYALWH are lost. The survey-plus-EMR analysis evaluated several additional variables that are likely associated with adolescent LTFU [48] to determine whether their inclusion could improve tool performance. These included depressive symptoms, alcohol and other drug use, social support, peer support group attendance, and exposure to violence. The final full model included PHQ-9 score, peer support group attendance, and any unmet clinical need at the time of clinic visit. The EMR-alone tool included two variables from EMR, age group and time in care. Including age-group improved performance of the EMR-alone tool although not the survey-plus-EMR tool. One reason could be that the EMR-alone tool had different (and fewer) predictors than the survey-plus-EMR tool.

We found that older age group (20–24 years) and longer time in care (six months or more) were predictors of LTFU, and older age has been shown to be associated with LTFU in prior studies [2]. Young adults may have higher risk of LTFU compared to adolescents because of less caregiver support to adhere to clinic visits or life changes, including marriage or seasonal travel. Longer time in care may have been a marker of AYALWH who had recently switched to multi-month ART refills. This change to their visit routine may have resulted in LTFU. Both versions of the tool were parsimonious, including fewer than five variables that are relatively easy to obtain and simple scores ranging from 0 to 2 or 4.

Compared to other clinical prediction tools developed for HIV prevention and care in sub-Saharan Africa, our tool included fewer variables and demonstrated lower performance by AUC. The ability of our tool to discriminate between AYALWH who were lost and not lost in both the training and validation cohorts ranged from 0.61 to 0.66, which is lower than the commonly used threshold of 0.7 [29]. Other tools developed to predict risk of HIV infection [33], HIV incidence among women [27, 28] and female adolescents [38, 49] and risk of viral non-suppression among adults [49] report performance by AUC ranging from 0.69 [27, 33, 37] to >0.90 [38]. One study among AYALWH enrolled in care in South Africa developed and validated a clinical prediction tool to determine readiness to transition to adult care, using the outcome of viral suppression [50]. This tool showed good performance across measures (e.g. AUC of 0.84) and included six predictors (ART regimen line, gender, HIV status disclosure, HIV Adolescent Readiness to Transition Scale score [51], age at ART initiation, and prior alcohol use). Direct comparisons of prediction tools are challenging because prediction models are highly sensitive to the variables included, variable section methods, and distribution of characteristics in the underlying population [34, 52]. In addition, our study outcome of LTFU measures clinic attendance and is not a direct proxy for ART adherence or viral non-suppression.

Other HIV-related prediction tools with performance of <0.7 by AUC have been evaluated in different research cohorts [37, 38] with intended use in routine care [37, 53]. For example, Puttkammer *et al*. [31] developed a prediction tool of ART failure among adults in HIV care in Haiti using the national EMR data, which had an AUC of 0.61. The authors then evaluated the feasibility of an intervention to improve viral suppression among adults with HIV in Haiti that combined an EMR-based alert system using the prediction tool to identify clients at risk of treatment failure and brief adherence counseling [53]. The intervention was feasible and acceptable, and associated with significantly improved adherence and a non-significant increased trend in viral suppression compared to baseline. However, providers reported challenges using the EMR-based tool including imperfect understanding of the alert criteria and mismatch between what the alert showed and how the client felt at the visit, which could raise doubts about the tool. Using prediction tools with sub-optimal performance (AUC <0.7) risk incorrectly identifying clients as needing a specific intervention or missing clients who do. However, as the study by Puttkammer *et al*., highlights, prediction tools with modest performance can offer useful information about challenges of implementing prediction tools into practice as well as other HIV service and client factors that need to be addressed for decision-support interventions in HIV care. Given the modest performance of both tools in our study, we do not recommend adapting these tools for routine care given the potential of mis-identifying AYALWH at risk of LTFU. Instead, the predictors evaluated in survey-plus-EMR and EMR-alone tools could be used as the basis for development of new prediction tools in similar populations and inform potential targets of future decision-support interventions with AYALWH.

Our study had strengths and limitations. To our knowledge, this is the first study to develop and validate a clinical prediction tool to identify AYALWH at risk of LTFU. We assessed psychosocial and service-related variables that have not been tested in prior prediction models for LTFU, and we considered both survey-plus-EMR and EMR-alone versions. The EMR-alone tool was limited to variables in that dataset, which were primarily clinical and demographic variables. LTFU using EMR data is subject to misclassification due to variable data quality and completeness [12]. We tried to minimize the possibility by using a definition of LTFU that aligns with the definition that facilities use, which accounted for more staggered visit schedules for clients in differentiated care. However, we could not verify whether some AYALWH we classified as LTFU had transferred out or were receiving care at another facility temporarily since EMRs are not synchronized across facilities. The survey-plus-EMR tool may have been underpowered to evaluate predictors with prevalence less than 10% in the sample, including harmful alcohol use and drug use, which may also be associated with LTFU in adolescent populations [50]. We also had to exclude outcomes after the initiation of COVID-19 response measures in Kenya, which reduced both sample size and follow-up time. The prevalence of LTFU in the survey-plus-EMR tool cohort was substantially lower than in the EMR-alone cohort, which likely reflected selection bias of AYALWH who enrolled in the survey when they came for clinic visits. In addition, there was a higher proportion of females and AYALWH ages 20–24 years in the EMR-alone compared to the survey-plus-EMR tool. The prevalence of LTFU in the survey-plus-EMR tool cohort was substantially lower than in the EMR-alone cohort, which likely reflected selection bias of AYALWH who enrolled in the survey. In addition, there was a higher proportion of females and AYALWH ages 20–24 years in the EMR-alone compared to the survey-plus-EMR tool. It is possible that the number and type of client-level predictors and associations with LTFU may differ in more urban settings. Differences in samples limited direct comparisons between tools. However, by using both data sources, we could evaluate a larger cohort (EMR-alone) that was generalizable to the clinic population and a sample that included behavioral survey data that are not captured in the EMR. New probabilistic methods, specifically Machine Learning, are being applied to clinical prediction models in HIV research [52]. While the data and technical requirements to use Machine Learning were outside the scope of this study, future studies could evaluate the feasibility of using this approach for prediction modeling in routine clinic settings [54, 55]. Finally, decisions about whether and how to use a prediction tool in routine practice depend on multiple considerations, including stakeholder engagement in intervention development, available services, and data systems [35] and qualitative and/or quantitative assessments of clinical usefulness [56–59].

## Conclusions

In summary, our clinical prediction tools using surveys-plus-EMR or EMR-alone showed modest predictive ability to identify AYALWH at risk of loss to follow-up and would have limited use to improve clinical decision making in this population. Accurately predicting LTFU among AYALWH remains challenging, especially as national guidelines change about visit timing for AYALWH change. Regardless, these results offer insights for future provider decision-support interventions to reduce LTFU among AYALWH clients, specifically the importance of including standard mental health screening and peer support services with this population.
